# Novel Blood-Based Biomarkers and Disease Modifying Therapies for Alzheimer's Disease. Are We Ready for the New Era?

**DOI:** 10.14283/jpad.2024.83

**Published:** 2024-05-21

**Authors:** Roxanna Korologou-Linden, J. Kalsi, D. Kafetsouli, A. Olawale, D. Wingfield, D. Mummery, B. Hayhoe, O. Robinson, A. Majeed, L.T. Middleton

**Affiliations:** 1 Ageing Epidemiology (AGE) Research Unit, School of Public Health, Imperial College London, 11th Floor, Charing Cross Hospital Campus, W6 8RP, London, UK; 2 Department of Primary Care and Public Health, Imperial College London, London, UK; 3 Department of Brain Sciences, Imperial College London, London, UK; 4 NIHR Applied Research Collaboration Northwest London, London, UK; 5 MRC Centre for Environment and Health, Department of Epidemiology and Biostatistics, School of Public Health, Imperial College London, London, UK; 6 Public Health Directorate, Imperial College Healthcare NHS Trust, London, UK

**Keywords:** Alzheimer's disease, blood-based biomarkers, monoclonal antibody therapies, Lecanemab, Donanemab

## Abstract

Recent positive trials for novel disease modifying therapies of anti-amyloid monoclonal antibodies represent a paradigm shift in the prevention and management of Alzheimer's disease, a relentlessly progressive and debilitating disease of old age. The reported efficacy of these new agents when given early in the disease trajectory is dependent on an early and accurate disease diagnosis, which is currently based on cerebrospinal fluid tests or/and neuro-imaging studies such as positron emission tomography. These confirmatory tests provide in vivo evidence of the pathological signature of Alzheimer's disease, of increased cerebral amyloid and tau burden and neurodegeneration. The emergence of blood-based biomarkers represents another breakthrough, offering a less invasive and scalable diagnostic tool that could be applied in both primary and specialist care settings, potentially revolutionizing Alzheimer's disease clinical pathways. However, healthcare systems face challenges in the adoption of these new technologies and therapies due to diagnostic and treatment capacity constraints, as well as financial and infrastructure requirements.

## Introduction

**G**lobally, dementia is the seventh leading cause of mortality, with the lives of approximately 55 million individuals affected globally (1). The numbers of dementia sufferers are expected to rise further, due to the unprecedented increases of life expectancy, even if incidence and prevalence rates may remain stable or decrease (2, 3, 4).

It has been estimated that Alzheimer's disease (AD), with in-vivo or post-mortem evidence of associated pathological changes, represents around 90% of late-onset dementia cases (5), although mixed pathologies are not uncommon in the oldest old (6, 7). In addition to the number of people at the clinical phase of AD, there exists a similar number of people experiencing early symptoms of AD, and an even greater at-risk population in preclinical disease stages (8). Individuals in the latter group exhibit normal cognitive abilities yet carry biomarkers indicative of the presence of AD pathological features, placing them at a higher risk for future cognitive decline (9, 10). Despite the significant challenges posed by AD and related dementias (ADRD) to patients, healthcare systems and societies, only seven (mostly symptomatic) drugs have been approved for AD (11) since 2000, in stark contrast to over 200 for cancer (12). However, the recent success of disease modifying therapies (DMTs) in anti-amyloid monoclonal antibodies has re-invigorated research and development efforts towards effective AD therapies and has built hope for eventual future widespread use of DMTs in healthcare settings, worldwide.

The average duration of the clinical manifestation of AD is about 8–10 years (13). However, the onset of overt cognitive decline is preceded by a long pre-symptomatic period, possibly extending over twenty years, characterized by progressive pathological accumulation (10, 13, 14). In AD, the pre-clinical stage is associated with build-up of the typical pathological AD “triad” signature; initially soluble amyloid beta (Aβ) species and interstitial cerebral “amyloid plaques” (A), the phosphorylation and subsequent aggregation of Tau proteins into neurofilament tangles within neurons (T), with progressive loss of cerebral volume and brain atrophy and other features of neurodegeneration (N) (10, 13, 14).

## Current clinical diagnostic pathway

The current gold standard of diagnosis, besides postmortem pathological evaluation of AD, is based on in vivo evidence of increased Aβ and Tau brain burden, measured by either positron emission tomography (PET) scans and/or cerebrospinal fluid (CSF) studies (10). These allow for the ante-mortem identification of the amyloid, Tau, and neurodegeneration (ATN) “molecular and cellular signature” of AD pathobiological processes (10, 14). However, CSF sampling requires a lumbar puncture, which is often perceived as being an invasive procedure, with potential side effects; PET is rarely accessible due to its relatively high costs and challenges in tracer production whilst PET scans require exposure to radiation. Hence, these barriers have limited their use outside a small number of specialist centres, at least in Europe and the UK.

The clinical diagnostic pathway of dementia for older adults is, still, broadly based on evaluating clinical symptomatology and progression. When an individual, their relatives or carers are suspicious of dementia, they first usually attend a primary care service for their concerns about memory, mood or changes in personality. The physician will perform an initial clinical assessment and where the assessment suggests that dementia is a likely diagnosis, the patient will be referred to a memory clinic for further clinical evaluation, typically aimed at establishing the presence of cognitive decline and to identify cases of potentially reversible aetiology (15). Only a minority of patients are referred to specialist units for further investigations (16). Approximately 25%–30% of individuals with a clinical diagnosis of AD are misdiagnosed when evaluated at specialised clinics and this is even higher in primary care (17, 18, 19, 20). Furthermore, there are inequalities in diagnosis, with individuals from ethnic minority groups, that are single, and those with greater cognitive function, lower agitation, and functional impairment in activities of daily living, or those who were more physically fit more likely to be missed (21).

## Novel and promising treatments for delaying disease onset and progression

Novel DMTs for AD that have proven effective in delaying disease progression include pharmaceutical agents targeting disease-specific aetiologies such as Aβ and tau (22, 23). Furthermore, multi-domain lifestyle interventions targeting pathways associated with biological ageing such as oxidative stress and other metabolic and vascular factors (24) can also improve cognitive performance and have a lasting effect on dementia incidence and several other health outcomes, such as cardiovascular risk and multi-morbidity in older adults (25). Following over two decades of failures of pharmaceutical research and development towards DMTs in AD (9), there have been recent successes in trials of anti-amyloid monoclonal antibody-based therapies in early symptomatic phases of “mild cognitive impairment” (MCI) and early dementia due to AD (22, 23). Two of these, Lecanemab and Donanemab have been shown to significantly reduce the brain amyloid load and potentially delay further cognitive and functional decline and dementia, if administered in the early stages of the disease. Indeed, Donanemab has been shown to be less effective in amyloid positive individuals that have a high Tau burden (22, 26). Both drugs are currently being tested in secondary prevention trials, in cognitively unimpaired (CU) individuals at high risk of dementia, based on evidence of being positive for ATN (27, 28).

## Readiness of Healthcare Systems

There are significant challenges for the widespread use of monoclonal antibody therapies, including their high individual costs, amplified by the ever-increasing number of AD sufferers, low diagnosis rates, healthcare funding and infrastructure challenges.

The patient groups that may qualify for monoclonal antibody therapies mainly involve individuals in the prodromal disease stages or patients with early stages of MCI/early AD (22, 23, 29). However, if these treatments were to be administered to all eligible individuals in Europe at the same price as in the United States, the cost is estimated to be 133 billion euros annually (29). It has been suggested that the full recommended usage of monoclonal antibody therapies such as Lecanemab could lead to its costs representing more than half of all drug spending across the European Union, based on conservative estimates (29).

Furthermore, the implementation of these therapies prerequire securing larger diagnostic capacity, additional training for the healthcare workforce and public awareness of early symptoms of AD, as well as an increase in genetic testing capacity (30). Globally, about 75% of people with dementia remain undiagnosed in high income countries, with this estimate reaching 90% in low and middle-income countries (17, 18, 19, 20, 31), with only a fraction of them having a specific AD diagnosis. Additionally, previous knowledge about APOE carriage had limited practical use and genetic testing was discouraged in suspected AD (32). However, amyloid-related imaging abnormalities (ARIA) have been observed on magnetic resonance imaging (MRI) scans after anti-amyloid immunotherapies (22, 23), with a higher prevalence in homozygous APOE ε4 genotype carriers (40.6%) compared to non-carriers (5.4%) (22). Hence, screening for the APOE genotype has become an integral part of the precise and personalized clinical and biological characterization for patient selection of such DMTs, due to the adverse interaction between monoclonal antibodies and the APOE ε4 genotype (32).

These therapies also require regular drug infusions and drug monitoring for side effects (e.g., ARIA) using sequential MRIs over the treatment course (33). Apart from being a gold standard pre-mortem diagnostic tool for AD, amyloid PET scans are required to accurately measure the treatment effect on reducing the cerebral amyloid burden, in conjunction to cognitive testing of clinical outcomes. However, amyloid or tau PET scanning are not presently recommended by NICE in the United Kingdom or by regulatory agencies and payers in Europe and other parts of the world, as there was little evidence of clinical value and cost-effectiveness prior to the emergence of these new therapies (34).

In summary, major changes to the infrastructure of the dementia care pathway need to take place before the potential roll-out of these therapies. The lack of resources for accurate AD diagnosis highlights the need for better diagnostic tools for easily accessible and inexpensive screening of individuals in primary care. Furthermore, the proviso for the introduction of AD monoclonal antibody therapies into the public healthcare system, is that they are affordable. This is will hopefully be the reality, as research and development efforts for additional pharmaceutical therapies of AD increase. Arguably, the highest barrier alongside its high cost is catching the disease early in its course.

## The implementation of blood-based biomarkers in primary and secondary care settings

The emerging evidence for an in vivo biomarker-based confirmatory diagnosis of the pathological ATN signature of AD prompted a research framework by the National Institute on Ageing and Alzheimer's Association in 2018 based on ATN for clinical research, which shifted the definition of AD from a purely clinical to a biological construct (35). As such, the disease was defined and staged across its continuum, using underlying pathology, as reflected by imaging (PET and MRI) or CSF biomarkers, rather than solely based on clinical symptoms and signs of cognitive decline on validated multidomain cognitive batteries (35). As research progresses into blood-based biomarkers (BBMs), going beyond ATN to other putative components of the “aetio-pathogenic puzzle” of this complex and multi-factorial disease, the system is expanding toward ATX(N), where “X” represents other markers, such as those referring to inflammation, accelerated brain ageing, as well as metabolic, vascular and other factors (24, 36, 37). Furthermore, recently initiated large international collaborative efforts, using cutting-edge technologies, aim at expanding the biomarker pool for AD pathological signatures and, thus, potentially dissecting AD disease heterogeneity. One such initiative is the Global Neurodegeneration Proteomics Consortium (GNPC), which leads research into the proteomic fingerprinting of AD and other neurodegenerative disorders.

As our understanding of all the pieces of the puzzle and their respective personalized biomarker-based signatures and roles grows, the future of DMTs in AD may well follow the “combination therapy” model that has proven its value in the treatment of HIV and several common multifactorial diseases, such as cancer and hypertension (38).

Mass spectrometry-based (MS) and fully automated immunoassay methodologies in plasma samples are now emerging as being precise and robust, in clinical research studies (39). BBMs such as several Tau species (phosphorylated (p)-Tau 181, p-Tau 217, p-Tau 231, MTBR-tau243, and others), Aβ42/40, or algorithms combining biomarker data, APOE and age are already in use as pre-screening tools in clinical trials to reduce the number of amyloid negative individuals before undergoing CSF or PET testing to confirm diagnosis of AD (27, 40). One such example is a commercially available plasma test which uses an algorithm, incorporating plasma Aβ42/40, APOE, Tau (p-Tau217/np-Tau217) and age to generate the amyloid probability score (APS) and has been validated for its diagnostic use in estimating the likelihood of being positive for amyloid on a PET scan. The area under the curve (AUC) for this test with the inclusion of p-Tau ratio was 95% and its accuracy 88%, when validated in two independent cohorts (41). Furthermore, the plasma p-Tau217 levels can also identify amyloid positive and negative individuals with high accuracy and may be a useful pre-screening tool to filter for eligible participants with high amyloid burden on PET or CSF in anti-amyloid immunotherapy trials (26).

Plasma biomarkers haven demonstrated their diagnostic and prognostic value in AD patients and CU at-risk individuals respectively, even before exceeding the amyloid PET and CSF positivity thresholds (39, 44, 45, 46, 47). Both the Aβ42 and Aβ42/40 ratio are predictive of amyloid pathology in the brain (48, 49, 50, 51); however, the fold change in these measures between Aβ+ and Aβ− individuals has been shown to be significantly smaller in plasma than in CSF, which limits its specificity in routinely identifying underlying AD pathology (50, 51). These concerns are further compounded by the inter-assay coefficient of variability (52), which largely affects the diagnostic accuracy. Plasma Tau biomarkers have been shown to be more valuable in accurately representing the disease continuum and the heterogeneity within the AD phenotype (43, 46, 53, 54).

In the last five years, there have been many studies reporting the high accuracy of plasma tau biomarkers in diagnosing AD (43, 45, 46, 55, 56, 57, 58), differentiating AD from other neurodegenerative diseases (51, 53, 55, 59, 60), and predicting future dementia (50, 55, 59, 61). Furthermore, plasma tau biomarkers have also been validated with neuropathological confirmation (51, 61, 62, 63, 64, 65). Plasma %p-Tau217 (ratio of phosphorylated-Tau217 to non-phosphorylated Tau217), measured through mass spectrometry assays, demonstrated clinical equivalence to US-approved CSF biomarkers in the classification of tau PET status (AUC=0.95–0.97) and superiority in the classification of Aβ PET status (AUC= 0.95–0.98) in two independent cohorts (Swedish BioFINDER-2 cohort (N=1,422) and US Knight ADRC (N=337)) (43). Furthermore, in cognitively impaired sub-cohorts, the positive predictive value of plasma %p-Tau217 was equivalent to the CSF tests (43). Thus, based on this emerging evidence, a simple blood test can detect AD with the same or even greater accuracy than the alternative gold standard of CSF and PET biomarkers of amyloid and tau.

Additionally, the pTau217 biomarker may be a surrogate marker for disease progression, which will be useful in monitoring the efficacy of the novel DMTs. In the BioFINDER-1 cohort of individuals who had MCI or were cognitively unimpaired (NCU=147, NMCI=95) (66), p-Tau217 showed clear changes in Aβ+ individuals compared to Aβ− individuals in both pre-clinical and symptomatic AD stages over a span of four to six years. The trajectory of plasma p-Tau 217 was also correlated with changes in cognitive domains as well as brain atrophy in regions typically implicated in AD (66). In another study using the same cohort, plasma P-tau 217 was shown to predict progression to AD with high accuracy (AUC=83%), which increased with the addition of memory, executive function, and APOE data (AUC=91%). This study showed that the accuracy of plasma biomarkers in research settings seems to now be comparable to CSF p-Tau, Aβ42/Aβ40 and neurofilament light chain values and is significantly greater than the clinical predictions made by doctors in memory clinics, using cognitive tests and structural brain imaging (4-year AUC=0.71) (54).

## Requirements for the implementation of blood-based biomarkers in primary and secondary care settings

AD plasma biomarkers have not yet been implemented on their own merit as a single diagnostic tool in clinical practice, due to limited evidence of their equivalence to CSF and PET results in real world clinical settings (39, 42, 43). For a biomarker to be scalable from research settings to clinical practice, it needs to be validated prospectively with scientific rigour in real-world clinical settings, encompassing both primary care and specialist memory centres. This is a necessary step to ensure its analytical and clinical robustness in diverse populations, proving its generalizability in populations with different demographic and other characteristics, before it is introduced to the market. Another requirement is for pre-determined thresholds to demonstrate clinical utility, independent of variations in sample handling, operators and laboratories. Hence, future research should focus on prospective validation in real world clinical settings and the inclusion of more diverse populations for increased generalizability of findings, as current studies are mainly based on retrospective cohorts from specialised centres (39). Piloting efforts to test the real-world implementation of BBMs include those by the Davos Alzheimer's Collaborative (DAC) SP Accurate Diagnosis Project, and the AD RIDDLE study, the latter being funded by the EU Innovative Health Initiative (IHI) and UK Research and Innovation (UKRI) (67).

Most importantly, the current commercial assays are at prohibitive costs and rely on complex technologies, posing challenges for large-scale provision in clinical practice worldwide. Given the accumulating positive results about their high specificity and sensitivity, developers seem responsive to their potential value in diagnostic and care pathways and are already taking the necessary steps for these technologies to be successfully transferable to clinical practice within the next two to five years, assuming their regulatory approvals within this and next year. It is not unreasonable to predict the future BBM assays to be the equivalent in ease and costs of current “routine” laboratory tests.

BBMs can work synergistically with ecologically valid, accessible and low-threshold digital tools assessing clinical cognitive and behavioural status, to facilitate an early, precise and personalized diagnosis, as well as track longitudinal disease trajectories and treatment responses (68).

In conclusion, based on the available evidence from recent research studies, the latest BBMs are highly promising AD diagnostic tools that are potentially more easily accessible, and which can be used at scale in primary care settings and may thus be useful for creating time and cost-effective patient-centred diagnostic and treatment plans. Given the relative unaffordability and invasiveness of the established CSF and PET biomarker tests, BBMs can play a key role in accelerating and scaling up the diagnostic pathways of early symptomatic AD stages and prevention strategies in at risk CU individuals. Additionally, they will aid in facilitating the implementation of novel current and future DMT therapies, whose clinical efficacy is expected to be optimal before irreversible neurodegeneration has begun. The AD field is rapidly moving into an entirely new era of answers and solutions, rather than unanswered questions. Therefore, this serves as an urgent call for transformation in diagnostic and care pathways, training of healthcare professionals, investment in infrastructure and appropriate funding, to allow for the adoption of new technologies and emerging novel DMTs. There is no time to waste.

*Funding:* This research was funded in part by the National Institute for Health and Care Research (NIHR) under the NIHR Applied Research Collaboration (ARC) programme for North-West London and the NIHR Imperial Biomedical Research Centre. The views expressed in this publication are those of the authors and not necessarily those of the NHS, the NIHR or the Department of Health and Social Care.

*Conflicts of Interest:* Dr. Middleton reports grants from Johnson & Johnson, Merck, Takeda, Gates Ventures, Eisai, NIHR, UKRI, and research funding from the Davos Alzheimer's Collaborative (DAC), outside the submitted work; all to Imperial College London. Dr. Wingfield reports research funding from NIHR and DAC, outside the submitted work. All other authors have no conflicts of interest to disclose.

## References

[1] World Health Organization (2023 Mar). Fact sheets of dementia. https://www.who.int/news-room/fact-sheets/detail/dementia.

[2] (2020 Mar 1). 2020 Alzheimer's disease facts and figures. Alzheimer's and Dementia.

[3] Wu YT, Beiser AS, Breteler MMB, Fratiglioni L, Helmer C, Hendrie HC (2017). The changing prevalence and incidence of dementia over time - current evidence. Nat Rev Neurol.

[4] Prince M, Ali GC, Guerchet M, Prina AM, Albanese E, Wu YT (2016 Jul 30). Recent global trends in the prevalence and incidence of dementia, and survival with dementia. Alzheimers Res Ther.

[5] Galasko D, Hansen LA, Katzman R, Wiederholt W, Masliah E, Terry R (1994). Clinical-neuropathological correlations in Alzheimer's disease and related dementias. Arch Neurol.

[6] Jellinger KA (2022). Recent update on the heterogeneity of the Alzheimer's disease spectrum. J Neural Transm.

[7] James BD, Bennett DA, Boyle PA, Leurgans S, Schneider JA (2012). Dementia From Alzheimer Disease and Mixed Pathologies in the Oldest Old. JAMA.

[8] Gustavsson A, Norton N, Fast T, Frölich L, Georges J, Holzapfel D (2023). Global estimates on the number of persons across the Alzheimer's disease continuum. Alzheimer's and Dementia.

[9] Cummings J, Zhou Y, Lee G, Zhong K, Fonseca J, Cheng F (2023). Alzheimer's disease drug development pipeline: 2023. Alzheimer's & Dementia: Translational Research & Clinical Interventions.

[10] Scheltens P, De Strooper B, Kivipelto M, Holstege H, Chételat G, Teunissen CE (2021). Alzheimer's disease. Lancet.

[11] CenterWatch Alzheimer's Disease ∣ FDA Approved Drugs, n.d. https://www.centerwatch.com/directories/1067-fda-approved-drugs/topic/137-alzheimer-s-disease.

[12] CenterWatch Oncology ∣ FDA Approved Drugs, n.d. https://www.centerwatch.com/directories/1067-fda-approved-drugs/topic/103-oncology.

[13] Masters CL, Bateman R, Blennow K, Rowe CC, Sperling RA, Cummings JL (2015).

[14] Jack CR, Knopman DS, Jagust WJ, Petersen RC, Weiner MW, Aisen PS (2013). Tracking pathophysiological processes in Alzheimer's disease: an updated hypothetical model of dynamic biomarkers. Lancet Neurol.

[15] Hayhoe B, Majeed A, Perneczky R (2016). General practitioner referrals to memory clinics: are referral criteria delaying the diagnosis of dementia?. J R Soc Med.

[16] Royal College of Psychiatrists (2018). https://www.rcpsych.ac.uk/improving-care/nccmh/service-design-and-development/dementia#:~:text=The%20dementia%20care%20pathway%3A%20full,and%20their%20families%20and%20carers.

[17] Rizzo G, Arcuti S, Copetti M, Alessandria M, Savica R, Fontana A (2018). Accuracy of clinical diagnosis of dementia with Lewy bodies: a systematic review and meta-analysis. J Neurol Neurosurg Psychiatry.

[18] Hansson O, Edelmayer RM, Boxer AL, Carrillo MC, Mielke MM, Rabinovici GD (2022). The Alzheimer's Association appropriate use recommendations for blood biomarkers in Alzheimer's disease. Alzheimer's & Dementia.

[19] Knopman DS, DeKosky ST, Cummings JL, Chui H, Corey-Bloom J, Relkin N (2001). Practice parameter: diagnosis of dementia (an evidence-based review). Report of the Quality Standards Subcommittee of the American Academy of Neurology. Neurology.

[20] Beach TG, Monsell SE, Phillips LE, Kukull W (2012). Accuracy of the Clinical Diagnosis of Alzheimer Disease at National Institute on Aging Alzheimer's Disease Centers, 2005–2010. J Neuropathol Exp Neurol.

[21] Sommerlad A, Perera G, Singh-Manoux A, Lewis G, Stewart R, Livingston G (2018). Accuracy of general hospital dementia diagnoses in England: Sensitivity, specificity, and predictors of diagnostic accuracy 2008–2016. Alzheimer's & Dementia.

[22] Sims JR, Zimmer JA, Evans CD, Lu M, Ardayfio P, Sparks JD (2023). Donanemab in Early Symptomatic Alzheimer Disease: The TRAILBLAZERALZ 2 Randomized Clinical Trial. JAMA.

[23] van Dyck CH, Swanson CJ, Aisen P, Bateman RJ, Chen C, Gee M (2022). Lecanemab in Early Alzheimer's Disease. New England Journal of Medicine.

[24] Hampel H, Cummings J, Blennow K, Gao P, Jack CR, Vergallo A (2021). Developing the ATX(N) classification for use across the Alzheimer disease continuum. Nature Reviews Neurology.

[25] Lehtisalo J, Rusanen M, Solomon A, Antikainen R, Laatikainen T, Peltonen M (2022). Effect of a multi-domain lifestyle intervention on cardiovascular risk in older people: the FINGER trial. Eur Heart J.

[26] Mattsson-Carlgren N, Collij LE, Stomrud E, Pichet Binette A, Ossenkoppele R, Smith R (2024). Plasma Biomarker Strategy for Selecting Patients With Alzheimer Disease for Antiamyloid Immunotherapies. JAMA Neurol.

[27] Rafii MS, Sperling RA, Donohue MC, Zhou J, Roberts C, Irizarry MC (2023). The AHEAD 3–45 Study: Design of a prevention trial for Alzheimer's disease. Alzheimer's & Dementia.

[28] (2024 Mar). ClinicalTrials.gov. A Donanemab (LY3002813) Prevention Study in Participants With Alzheimer's Disease (TRAILBLAZER-ALZ 3). https://classic.clinicaltrials.gov/ct2/show/NCT05026866.

[29] Jönsson L, Wimo A, Handels R, Johansson G, Boada M, Engelborghs S (2023 Jun 1). The affordability of lecanemab, an amyloid-targeting therapy for Alzheimer's disease: an EADC-EC viewpoint. The Lancet Regional Health - Europe.

[30] NHS England (2023 Dec). Preparing for a new chapter: disease modifying treatments for early Alzheimer's disease. https://www.england.nhs.uk/blog/preparing-for-a-new-chapter-disease-modifying-treatments-for-early-alzheimers-disease/#:~:text=NHS%20England%20has%20been%20closely,in%20the%20US%20and%20Japan.

[31] Gauthier S R-NPMJWC. World Alzheimer Report 2021. Journey through the diagnosis of dementia. London, England; 2021. Chrome-extension://efaidnbmnnnibpcajpcglclefindmkaj/https://www.alzint.org/u/World-Alzheimer-Report-2021.pdf. Accessed 23rd January 2024.

[32] Ronay S, Tsao JW (2024 Apr). The Importance of Apolipoprotein E Genetic Testing in the Era of Amyloid Lowering Therapies. Neurol Clin Pract.

[33] Barakos J, Purcell D, Suhy J, Chalkias S, Burkett P, Grassi CM (2022). Detection and Management of Amyloid-Related Imaging Abnormalities in Patients with Alzheimer's Disease Treated with Anti-Amyloid Beta Therapy. J Prev Alzheimers Dis.

[34] NICE (2018 Jun). Dementia: assessment, management and support for people living with dementia and their carers. https://www.nice.org.uk/guidance/ng97.

[35] Jack CR, Bennett DA, Blennow K, Carrillo MC, Dunn B, Haeberlein SB (2018). NIA-AA Research Framework: Toward a biological definition of Alzheimer's disease. Alzheimer's and Dementia.

[36] Oh HSH, Rutledge J, Nachun D, Pálovics R, Abiose O, Moran-Losada P (2023). Organ aging signatures in the plasma proteome track health and disease. Nature.

[37] Guo Y, You J, Zhang Y, Liu W-S, Huang Y-Y, Zhang Y-R (2024). Plasma proteomic profiles predict future dementia in healthy adults. Nature Aging.

[38] Middleton LT, Touchon J, Vellas B (2023). What Is Reasonable and Necessary for Alzheimer Patients from Randomized Clinical Trials to Clinical Practice?. Journal of Prevention of Alzheimer's Disease.

[39] Angioni D, Delrieu J, Hansson O, Fillit H, Aisen P, Cummings J (2022). Blood Biomarkers from Research Use to Clinical Practice: What Must Be Done? A Report from the EU/US CTAD Task Force. J Prev Alzheimers Dis.

[40] Tariot P, Reiman E, Alexander R (2021). TRAILBLAZER-ALZ3 trial design and rationale. J Prev Alzheimer's Dis.

[41] Rissman RA, Langford O, Raman R, Donohue MC, Abdel-Latif S, Meyer MR (2023). Plasma Aβ42/Aβ40 and phospho-tau217 concentration ratios increase the accuracy of amyloid PET classification in preclinical Alzheimer's disease. Alzheimer's & Dementia.

[42] Hampel H, Hu Y, Cummings J, Mattke S, Iwatsubo T, Nakamura A (2023). Blood-based biomarkers for Alzheimer's disease: Current state and future use in a transformed global healthcare landscape. Neuron.

[43] Barthélemy NR, Salvadó G, Schindler S, He Y, Janelidze S, Collij LE (2024 Feb 21). Highly Accurate Blood Test for Alzheimer's Disease Comparable or Superior to Clinical CSF Tests. Nature Medicine.

[44] Schindler SE, Bollinger JG, Ovod V, Mawuenyega KG, Li Y, Gordon BA (2019). High-precision plasma β-amyloid 42/40 predicts current and future brain amyloidosis. Neurology.

[45] Janelidze S, Palmqvist S, Leuzy A, Stomrud E, Verberk IMW, Zetterberg H (2022). Detecting amyloid positivity in early Alzheimer's disease using combinations of plasma Aβ42/Aβ40 and p-tau. Alzheimer's and Dementia.

[46] Janelidze S, Berron D, Smith R, Strandberg O, Proctor NK, Dage JL (2021). Associations of Plasma Phospho-Tau217 Levels With Tau Positron Emission Tomography in Early Alzheimer Disease. JAMA Neurol.

[47] Udeh-Momoh C, Zheng B, Sandebring-Matton A, Novak G, Kivipelto M, Jönsson L (2022). Blood Derived Amyloid Biomarkers for Alzheimer's Disease Prevention. Journal of Prevention of Alzheimer's Disease.

[48] Janelidze S, Teunissen CE, Zetterberg H, Allué JA, Sarasa L, Eichenlaub U (2021). Head-to-Head Comparison of 8 Plasma Amyloid-β 42/40 Assays in Alzheimer Disease. JAMA Neurol.

[49] Nakamura A, Kaneko N, Villemagne VL, Kato T, Doecke J, Doré V (2018). High performance plasma amyloid-β biomarkers for Alzheimer's disease. Nature.

[50] Schindler SE, Bollinger JG, Ovod V, Mawuenyega KG, Li Y, Gordon BA (2019). High-precision plasma β-amyloid 42/40 predicts current and future brain amyloidosis. Neurology.

[51] Ashton NJ, Pascoal TA, Karikari TK, Benedet AL, Lantero-Rodriguez J, Brinkmalm G (2021). Plasma p-tau231: a new biomarker for incipient Alzheimer's disease pathology. Acta Neuropathol.

[52] Benedet AL, Brum WS, Hansson O, Karikari TK, Zimmer ER, Zetterberg H (2022). The accuracy and robustness of plasma biomarker models for amyloid PET positivity. Alzheimers Res Ther.

[53] Lantero Rodriguez J, Karikari TK, Suárez-Calvet M, Troakes C, King A, Emersic A (2020). Plasma p-tau181 accurately predicts Alzheimer's disease pathology at least 8 years prior to post-mortem and improves the clinical characterisation of cognitive decline. Acta Neuropathol.

[54] Palmqvist S, Tideman P, Cullen N, Zetterberg H, Blennow K, Stomrud E (2021). Prediction of future Alzheimer's disease dementia using plasma phosphotau combined with other accessible measures. Alzheimer's & Dementia.

[55] Janelidze S, Mattsson N, Palmqvist S, Smith R, Beach TG, Serrano GE (2020). Plasma P-tau181 in Alzheimer's disease: relationship to other biomarkers, differential diagnosis, neuropathology and longitudinal progression to Alzheimer's dementia. Nature Medicine.

[56] Karikari TK, Pascoal TA, Ashton NJ, Janelidze S, Benedet AL, Rodriguez JL (2020). Blood phosphorylated tau 181 as a biomarker for Alzheimer's disease: a diagnostic performance and prediction modelling study using data from four prospective cohorts. Lancet Neurol.

[57] Palmqvist S, Janelidze S, Quiroz YT, Zetterberg H, Lopera F, Stomrud E (2020). Discriminative Accuracy of Plasma Phospho-tau217 for Alzheimer Disease vs Other Neurodegenerative Disorders. JAMA.

[58] Simrén J, Leuzy A, Karikari TK, Hye A, Benedet AL, Lantero-Rodriguez J (2021). The diagnostic and prognostic capabilities of plasma biomarkers in Alzheimer's disease. Alzheimer's & Dementia.

[59] Palmqvist S, Janelidze S, Quiroz YT, Zetterberg H, Lopera F, Stomrud E (2020). Discriminative Accuracy of Plasma Phospho-tau217 for Alzheimer Disease vs Other Neurodegenerative Disorders. JAMA.

[60] Thijssen EH, La Joie R, Wolf A, Strom A, Wang P, Iaccarino L (2020). Diagnostic value of plasma phosphorylated tau181 in Alzheimer's disease and frontotemporal lobar degeneration. Nature Medicine.

[61] Lantero Rodriguez J, Karikari TK, Suárez-Calvet M, Troakes C, King A, Emersic A (2020). Plasma p-tau181 accurately predicts Alzheimer's disease pathology at least 8 years prior to post-mortem and improves the clinical characterisation of cognitive decline. Acta Neuropathol.

[62] Smirnov DS, Ashton NJ, Blennow K, Zetterberg H, Simrén J, Lantero-Rodriguez J (2022). Plasma biomarkers for Alzheimer's Disease in relation to neuropathology and cognitive change. Acta Neuropathol.

[63] Salvadó G, Ossenkoppele R, Ashton NJ, Beach TG, Serrano GE, Reiman EM (2023). Specific associations between plasma biomarkers and postmortem amyloid plaque and tau tangle loads. EMBO Mol Med.

[64] Murray ME, Moloney CM, Kouri N, Syrjanen JA, Matchett BJ, Rothberg DM (2022). Global neuropathologic severity of Alzheimer's disease and locus coeruleus vulnerability influences plasma phosphorylated tau levels. Mol Neurodegener.

[65] Grothe MJ, Moscoso A, Ashton NJ, Karikari TK, Lantero-Rodriguez J, Snellman A (2021). Associations of Fully Automated CSF and Novel Plasma Biomarkers With Alzheimer Disease Neuropathology at Autopsy. Neurology.

[66] Ashton NJ, Janelidze S, Mattsson-Carlgren N, Binette AP, Strandberg O, Brum WS (2022). Differential roles of Aβ42/40, p-tau231 and p-tau217 for Alzheimer's trial selection and disease monitoring. Nature Medicine.

[67] Malzbender K, Barbarino P, Ferrell PB, Bradshaw A, Brookes AJ, Díaz C (2024 Jan 30). Validation, Deployment, and Real-World Implementation of a Modular Toolbox for Alzheimer's Disease Detection and Dementia Risk Reduction: The AD-RIDDLE Project. Journal of Prevention of Alzheimer's Disease.

[68] Hampel H, Au R, Mattke S, van der Flier WM, Aisen P, Apostolova L (2022). Designing the next-generation clinical care pathway for Alzheimer's disease. Nature Aging.

